# Transcriptome analysis of genes and metabolic pathways associated with nicotine degradation in *Aspergillus oryzae* 112822

**DOI:** 10.1186/s12864-019-5446-2

**Published:** 2019-01-24

**Authors:** Chunjuan He, Yougui Huang, Peng Liu, Jianhuan Wei, Yirui Yang, Li Xu, Min Xiao

**Affiliations:** State Key Laboratory of Microbial Technology, National Glycoengineering Research Center, Shandong Provincial Key Laboratory of Carbohydrate Chemistry and Glycobiology, Shandong University, Qingdao, 266237 China

**Keywords:** Nicotine degradation, Demethylation pathway, Transcriptome analysis, Cytochrome P450 monooxygenase, Detoxification, Energy investment, Oxidative stress response, Transporter

## Abstract

**Background:**

Nicotine-degrading microorganisms (NDMs) have recently received much attention since they can consume nicotine as carbon and nitrogen source for growth. In our previous work, we isolated an efficient nicotine-degrading fungus *Aspergillus oryzae* 112822 and first proposed a novel demethylation pathway of nicotine degradation in fungi. However, the underlying mechanisms of the demethylation pathway remain unresolved. In the present study, we performed a comparative transcriptome analysis to elucidate the molecular mechanisms of nicotine tolerance and degradation in *A. oryzae* 112822.

**Results:**

We acquired a global view of the transcriptional regulation of *A. oryzae* 112822 exposed to nicotine and identified 4381 differentially expressed genes (DEGs) by nicotine treatment. Candidate genes encoding cytochrome P450 monooxygenases (CYPs), FAD-containing amine oxidase, molybdenum cofactor (Moco)-containing hydroxylase, and NADH-dependent and FAD-containing hydroxylase were proposed to participate in the demethylation pathway of nicotine degradation. Analysis of these data also revealed that increased energy was invested to drive nicotine detoxification. Nicotine treatment led to overproduction of reactive oxygen species (ROS), which formed intracellular oxidative stress that could induce the expression of several antioxidant enzymes, such as superoxide dismutase (SOD), catalase (CAT), and peroxiredoxin (Prx). Thioredoxin system was induced to restore the intracellular redox homeostasis. Several glutathione S-transferases (GSTs) were induced, most likely to participate in phase II detoxification of nicotine by catalyzing the conjugation of glutathione (GSH) to active metabolites. The toxin efflux pumps, such as the ATP-Binding Cassette (ABC) transporters and the major facilitator superfamily (MFS) transporters, were overexpressed to overcome the intracellular toxin accumulation. By contrast, the metabolic pathways related to cellular growth and reproduction, such as ribosome biogenesis and DNA replication, were inhibited by nicotine treatment.

**Conclusion:**

These results revealed that complex regulation networks, involving detoxification, transport, and oxidative stress response accompanied by increased energy investment, were developed for nicotine tolerance and degradation in *A. oryzae* 112822. This work provided the first insight into the metabolic regulation of nicotine degradation and laid the foundation for further revealing the molecular mechanisms of the nicotine demethylation pathway in filamentous fungi.

**Electronic supplementary material:**

The online version of this article (10.1186/s12864-019-5446-2) contains supplementary material, which is available to authorized users.

## Background

Nicotine is the most abundant tobacco alkaloid, accounting for 95% of the total alkaloid content and weighing to the extent of 1.5% of the dry mass in commercially used tobacco [1]. As a hypertoxic *N*-heterocyclic compound, nicotine can cross the biological membrane and blood-brain barrier [2], interacting with the nicotinic acetylcholine receptors in the nervous systems and causing psychedelic, nausea, hypertension, mydriasis, arrhythmia, convulsion, collapse, and even death at certain dose [3]. Annually, large quantities of toxic and hazardous tobacco wastes containing high concentration of nicotine are accumulated from tobacco industry [4], and nicotine has been detected in surface water, groundwater, and even bottled mineral water, which displays potential ecotoxicological risk [5–7]. In order to alleviate the serious environmental problems caused by nicotine-containing wastes, bioremediation with the advantage of high efficiency and sustainability has been considered as the most promising method, which employs nicotine-degrading microorganisms (NDMs) utilizing nicotine as carbon and nitrogen sources for their growth [8]. Several NDMs have been used to detoxify the tobacco wastes [9, 10], to reduce nicotine dependence of smokers with gradually reduced nicotine content in cigarettes [11], and to produce valuable pyridine derivatives for drug synthesis [12].

**Scheme 1:**
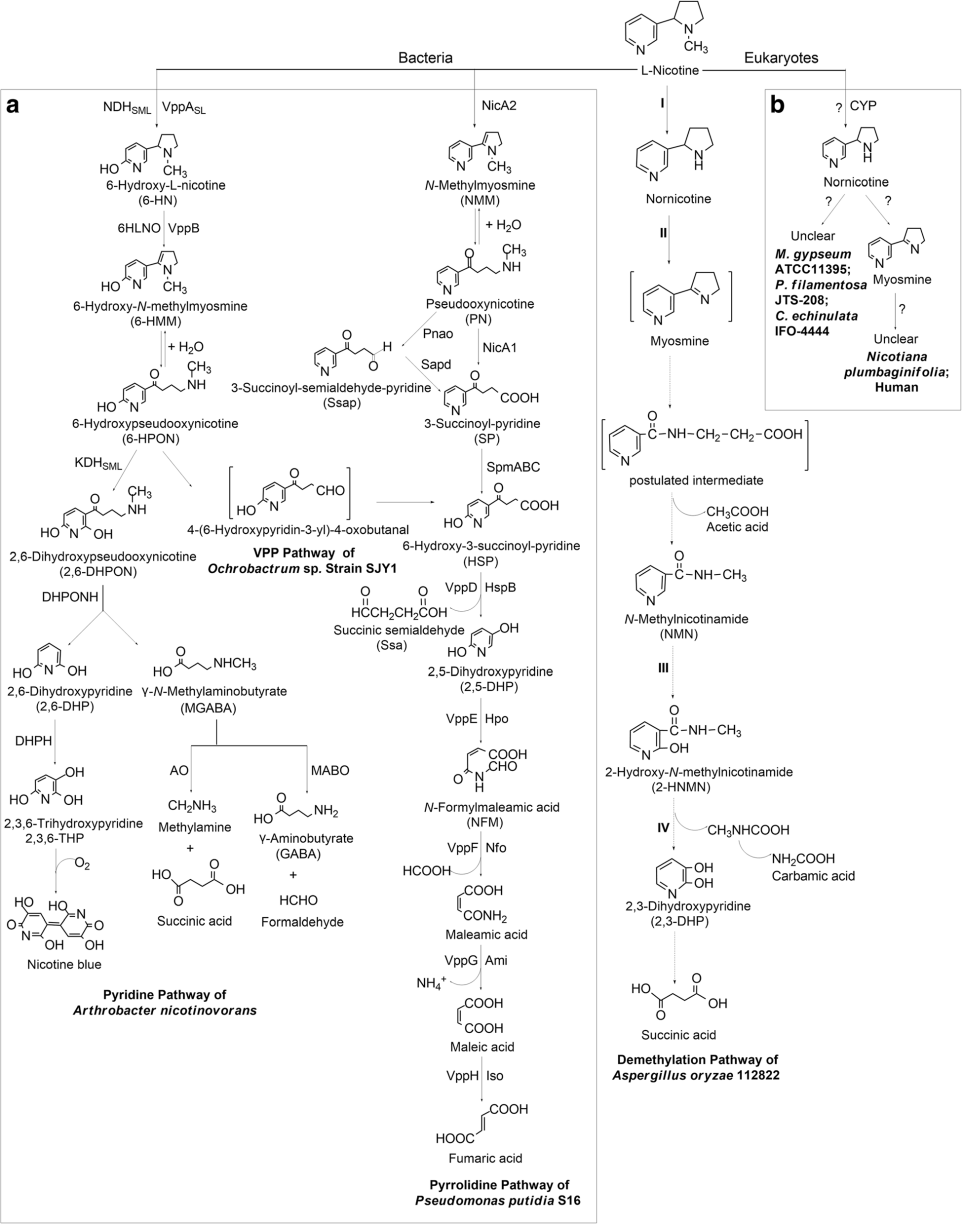
The reported nicotine degradation pathways in bacteria (**a**) and eukaryotes (**b**). Nicotine degradation pathways in bacteria are represented by the pyridine pathway of *A. nicotinovorans*, the pyrrolidine pathway of *P. putidia* S16, and the VPP pathway of *Ochrobactrum* sp*.* Strain SJY1; the demethylation pathways in eukaryotes are inordinately described for *A. oryzae* 112822, *M. gypseum* ATCC11395, *P. filamentosa* JTS-208, *C. echinulata* IFO-4444, *Nicotiana plumbaginifolia* and human. NDH_SML_, nicotine dehydrogenase; VppA_SL_, nicotine hydroxylase; 6HLNO and VppB, 6-HN oxidases; KDH_SML_, ketone dehydrogenase; DHPONH, 2, 6-dihydroxypseudooxynicotine hydrolase; DHPH, 2, 6-dihydroxypyridine 3-hydroxylase; AO, amine oxidase; MABO, γ-*N*-methylaminobutyrate oxidase; NicA1 and NicA2, nicotine oxidoreductases; Pnao, pseudooxynicotine amidase; Sapd, 3-succinoyl-semialdehyde-pyridine dehydrogenase; SpmABC, 3-succinoyl-pyridine monooxygenase; HspB and VppD, HSP 3-monooxygenases; Hpo and VppE, 2, 5-DHP dioxygenases; Nfo and VppF, *N*-formylmaleamic acid deformylases; VppG and Ami, maleamate amidases; Iso and VppH, maleate isomerases

Among these NDMs, several bacteria, such as *Arthrobacter nicotinovorans* [13]*, Pseudomonas putida* S16 [14]*, Pseudomonas* sp*.* HF-1 [15], *Pseudomonas* sp*.* HZN6 [16], *Ochrobactrum* sp*.* Strain SJY1 [17]*, Agrobacterium tumefaciens* S33 [18]*,* and *Shinella* sp*.* HZN7 [19] have been studied. Three nicotine degradation pathways have been fully elucidated in bacteria (Scheme 1). In the pyridine pathway, nicotine degradation begins with hydroxylation of the pyridine-ring to form 6-hydroxy-L-nicotine (6-HN) [13]. In the pyrrolidine pathway, nicotine is first converted into *N*-methylmyosmine (NMM) by dehydrogenation at the pyrrolidine-ring [14–16]. In the VPP pathway, nicotine is initially transformed into 6-hydroxypseudooxynicotine (6-HPON) through the pyridine pathway, and then the 6-HPON is further converted into 6-hydroxy-3-succinoyl-pyridine (HSP) which turns into the pyrrolidine pathway [17–19]. In bacteria, the enzymes involved in the first two pathways have been elucidated in detail [13, 14, 20–26], and several isoenzymes involved in the VPP pathway have also been reported [17, 27].

Compared with nicotine-degrading bacteria, few fungal strains, including *Microsporum gypseum* ATCC11395 [28], *Pellicularia filamentosa* JTS-208 [29], and *Cunninghamella echinulata* IFO-4444 [29] have been reported to have nicotine-degrading ability. All the three fungi degrade nicotine to form nornicotine via the *N*-demethylation reaction [28, 29]. Besides, the similar metabolite nornicotine, the downstream metabolite myosmine, and the enzyme catalyzing the conversion of nicotine to nornicotine have also been reported in the nicotine degradation process in tobacco [30–32] and human [33, 34] (Scheme 1). In our previous work, based on analysis of the metabolites, a detailed nicotine demethylation pathway was proposed in filamentous fungus *Aspergillus oryzae* 112822 [35]. In this pathway, nicotine is initially demethylated to form nornicotine which is further converted into myosmine by dehydrogenation at the pyrrolidine-ring. Subsequently, the cleavage of pyrrolidine-ring leads to the formation of a postulated intermediate metabolite which is converted into *N*-methylnicotinamide (NMN) by release of an acetic acid. NMN is hydroxylated to form 2-hydroxy-*N*-methylnicotinamide (2-HNMN) which degrades into a novel nicotine metabolite 2, 3-dihydroxypyridine (2, 3-DHP) with the formation of carbamic acid. The pyridine-ring cleavage of 2, 3-DHP results in formation of succinic acid which enters into tricarboxylic acid (TCA) cycle (Scheme 1). The first two reactions in this pathway resemble the initial nicotine degradation in tobacco [32] and human [34]. This demethylation pathway of *A. oryzae* 112822 is the first fully described nicotine degradation pathway in fungi, and it is significantly different from the three reported nicotine degradation pathways in bacteria. Several novel nicotine metabolites, such as NMN, 2-HNMN, and 2, 3-DHP are identified in this pathway [35]. However, related genes and enzymes involved in this demethylation pathway have not been revealed, and the underlying mechanisms remain unknown.

The genome sequence of *A. oryzae* RIB40 has become publically accessible, and the gene function and metabolic network have also been annotated based on developed annotation strategy [36]. In the present study, we employed a sequencing-based approach (RNA-Seq) to obtain a global view of the transcriptional regulation of *A. oryzae* 112822 in response to nicotine exposure. This work revealed that complex regulation networks, involving detoxification, transport, and oxidative stress response accompanied by increased energy investment, were developed for nicotine tolerance and degradation in *A. oryzae* 112822. Candidate genes that potentially participate in the demethylation pathway of nicotine degradation were screened out. These results could substantially provide a valuable genetic resource to explore the molecular mechanisms of the demethylation pathway in fungi.

## Results

### Analysis of nicotine degradation activity of *A. oryzae* 112822

**Fig. 1:**
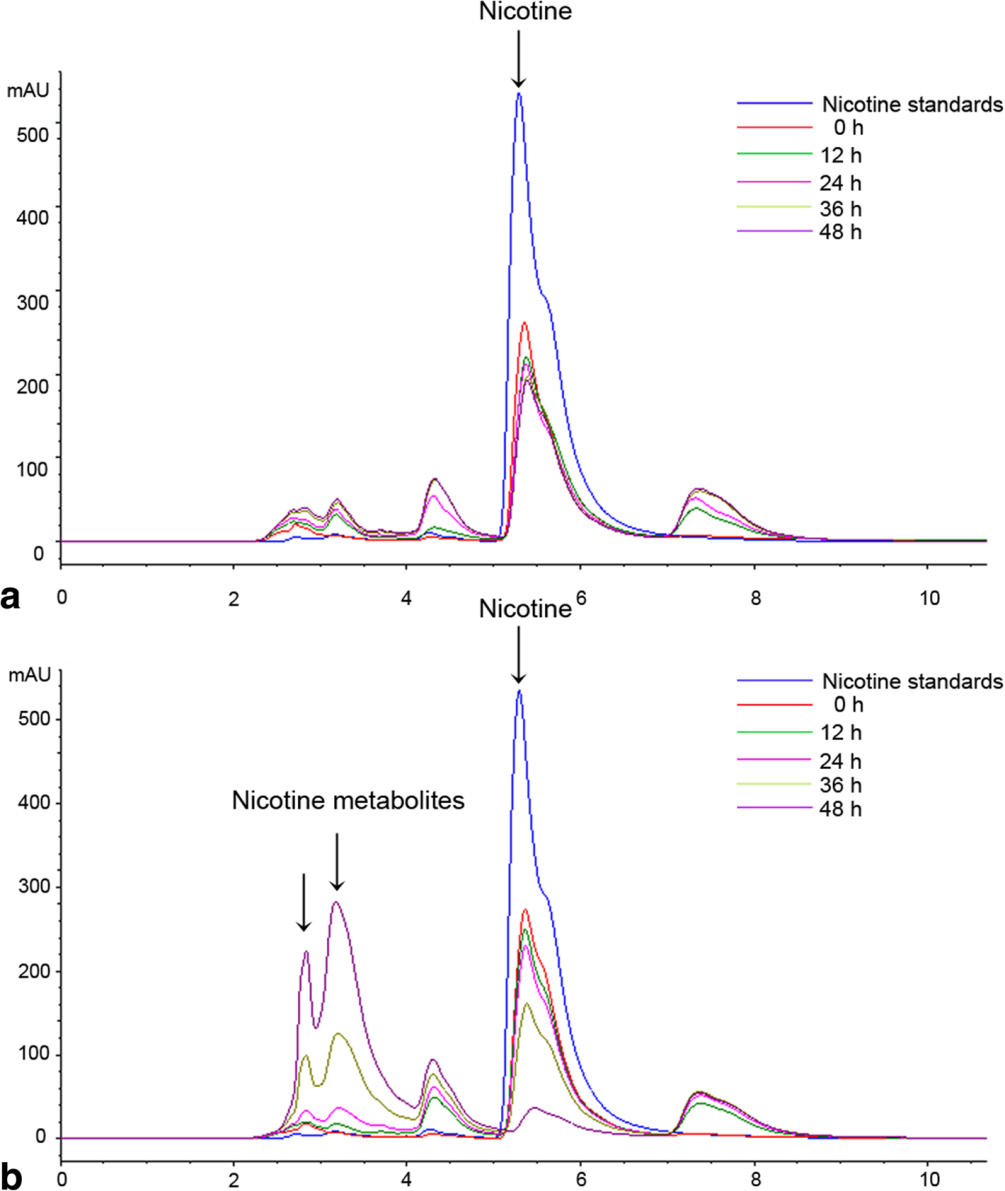
HPLC analysis of the nicotine degradation by resting cells of *A. oryzae* 112822. **a** The nicotine degradation by resting cells from dextrose-containing media. **b** The nicotine degradation by resting cells from nicotine-containing media. Reaction mixtures were sampled at regular intervals and analyzed on Agilent TC-C18 column with UV detector operating at a wavelength of 254 nm

The nicotine degradation activity of resting cells derived from *A. oryzae* 112822 cultured in dextrose-containing and nicotine-containing media was tested with 0.35 g·L^− 1^ nicotine in potassium phosphate buffer (50 mM, pH 6.5) at 28 °C. The residual nicotine was quantified using high performance liquid chromatography (HPLC). As shown in Fig. 1, nicotine was gradually consumed by 83% in 48 h by resting cells from nicotine-containing media, and several subsequent metabolites were generated during nicotine degradation. However, neither obvious nicotine degradation nor metabolites were detected in the reaction system constructed using resting cells from dextrose-containing media.

### RNA-Seq and assembly

Two groups of sequencing libraries were prepared from control (YD, *n* = 3) and nicotine-treated (Nic, n = 3) samples to investigate the transcriptional responses of *A. oryzae* 112822 to nicotine exposure. After filtering for quality control of the raw reads, an average of 44.72 Mb (75.21%) and 44.32 Mb (75.09%) clean reads with 6.71 Gb and 6.65 Gb clean bases, representing at least 180-fold of the reference genome of *A. oryzae* RIB40 (37 Mb) [36], were acquired from the YD and Nic libraries. These clean reads with approximately 97% Phred-like quality scores at the Q20 level were selected as high quality reads for further analysis. Then clean reads were mapped to reference genome with an average matching ratio of 84.01 and 86.89% for the YD and Nic libraries, respectively. A summary of the RNA-Seq and sequence mapping was presented in Table 1, which demonstrated the effectiveness for transcriptome analysis using the sequence-based method. As a result, 16,818 transcripts were reconstructed for all the sequencing libraries, 6096 novel transcripts were discovered by comparing with the reference annotation of *A. oryzae* RIB40, including 4639 novel splicing isoforms, 161 novel genes and 1296 novel non-coding transcripts (Table 2). Based on the alignment analysis, the coverage of the clean reads for transcripts suggested that approximately 70% of the transcripts displayed the coverage of 80–100% in the two groups of cDNA libraries (Additional file 1). These transcripts were assembled into 10,540 genes for YD and 10,308 genes for Nic with a union of 10,883 genes in total (Additional file 2), which accounted for approximately 90% of the annotated reference genome which contains 12,074 genes [36].

**Table 1:** Summary of sequencing and mapping results

Samples (−average)	Raw reads (Mb)	Clean reads (Mb)	Clean bases (Gb)	Clean reads Q20 (%)	Clean reads ratio (%)	Mapping ratio (%)	Uniquely mapping ratio (%)
YD1	57.15	45.19	6.78	97.88	79.06	84.49	66.38
YD2	57.15	44.87	6.73	97.86	78.51	83.90	65.93
YD3	64.78	44.09	6.61	96.88	68.06	83.63	60.43
YD-average	59.69	44.72	6.71	97.54	75.21	84.01	64.25
Nic1	55.52	44.78	6.72	97.91	80.66	87.42	58.59
Nic2	60.18	44.04	6.61	96.82	73.18	87.04	53.83
Nic3	61.80	44.15	6.62	96.87	71.43	86.20	53.67
Nic-average	59.17	44.32	6.65	97.20	75.09	86.89	55.36

**Table 2:** Summary of genes and transcripts

Genes	Known genes	Total transcripts	Novel transcripts	Novel coding transcripts	Novel isoforms	Novel genes	Novel noncoding transcripts
10,883	10,722	16,818	6096	4800	4639	161	1296

### Identification of differentially expressed genes (DEGs)

**Fig. 2:**
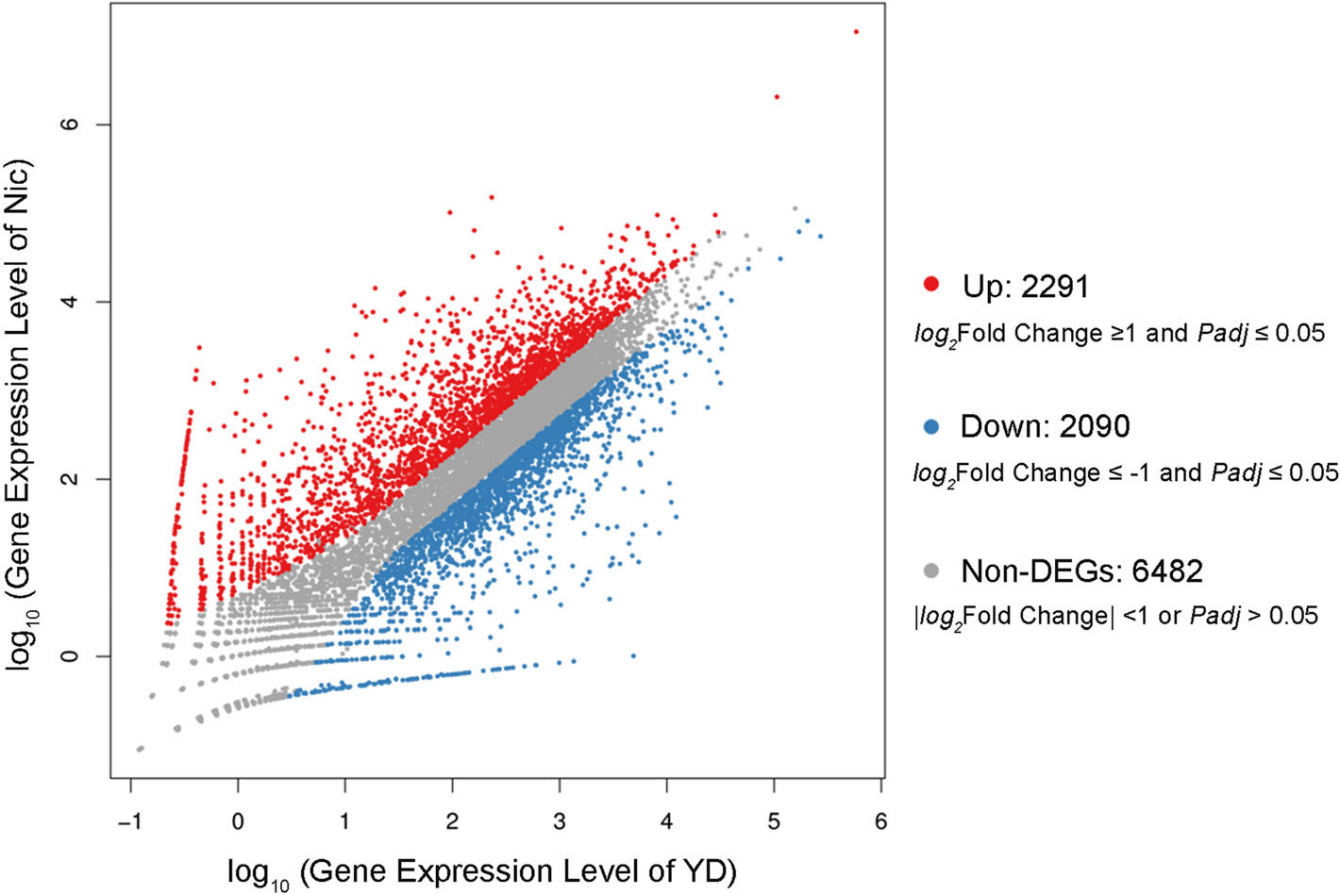
Scatter plot of DEGs between the YD and Nic libraries. Red spots represent the up-regulated DEGs, blue spots indicate the down-regulated DEGs, and gray spots represent the genes without significant differential expression

The transcript abundance of each gene was estimated by the RSEM software package [37] with 95% credibility intervals. As a result, 4381 genes showed significant difference in expression profiles [|log_2_ Fold Change| ≥ 1 and Adjusted *P*-value (Padj) ≤ 0.05], including 2291 up-regulated and 2090 down-regulated genes in the Nic library compared with those in the YD library (Fig. 2, Additional file 3: Table S1).

### Functional annotation, classification, and enrichment analysis

For functional annotation, all the assembled genes were searched against the National Center for Biotechnology Information (NCBI) non-redundant (Nr), Clusters of Orthologous Groups (COG), Gene Ontology (GO), and Kyoto Encyclopedia of Genes and Genomes (KEGG) databases using BLAST program with a cut-off E-value of 10^− 5^. Among the 10,883 genes, 10,070 (92.53%) genes had significant matches in the Nr database. Furthermore, COG, GO, and KEGG annotations were applicable for 6364 (58.48%), 7019 (64.50%), and 8536 (78.43%) genes, respectively (Table 3). Functional classification and enrichment analysis were further performed to identify the biological function of DEGs.

**Table 3:** Summary of functional annotations

Functional annotations	Assembled genes	DEGs
Total	10,883	4381
Nr	10,070 (92.53%)	4004 (91.39%)
COG	6364 (58.48%)	2476 (56.52%)
GO	7019 (64.50%)	2908 (66.38%)
KEGG	8536 (78.43%)	3269 (74.62%)

**Fig. 3:**
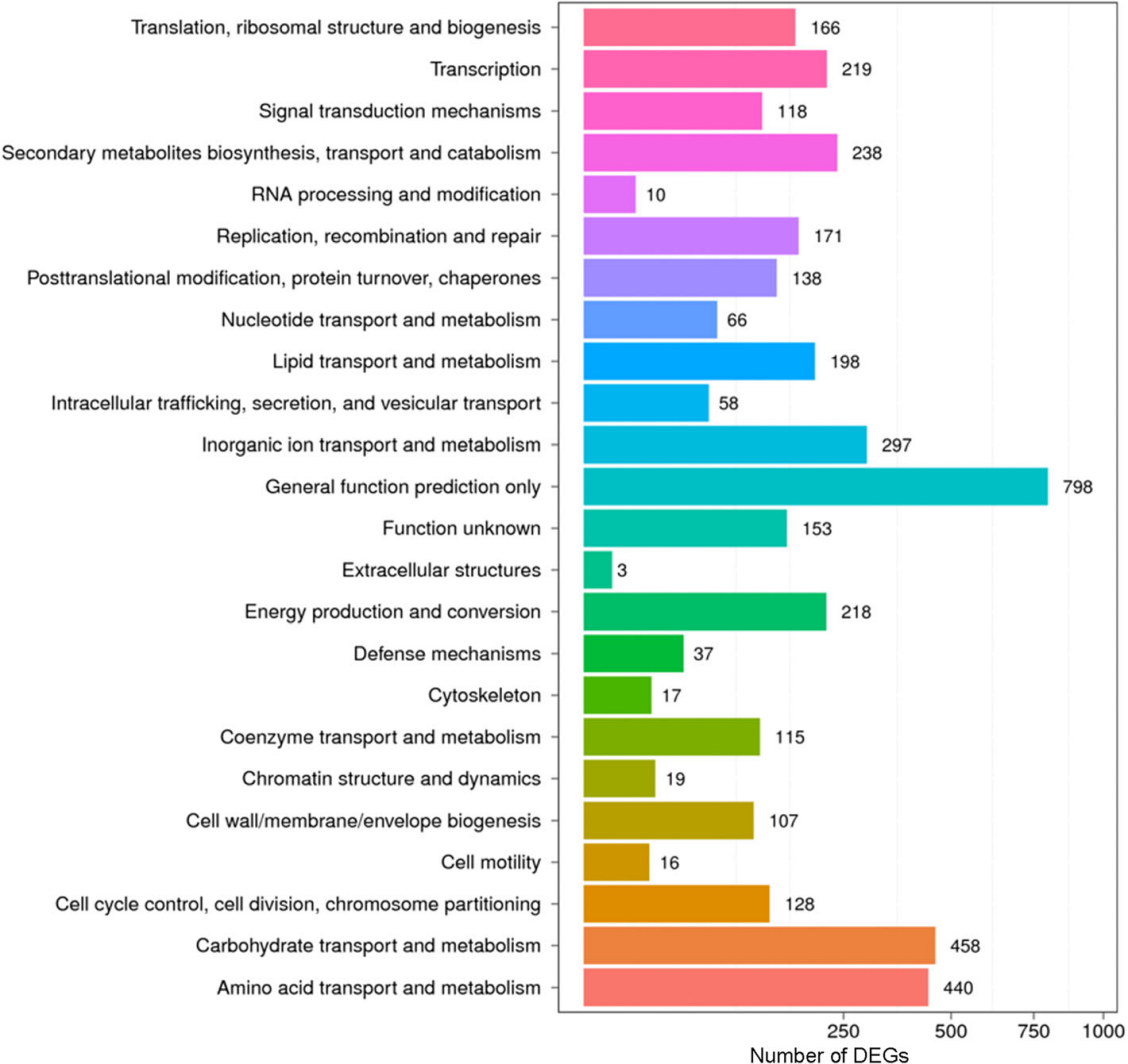
COG classification of DEGs. A total of 2476 DEGs showing significant homology to those in the COG database were functionally grouped into 24 specific categories. The x-axis indicates the number of DEGs, and the y-axis indicates the specific COG functional clusters

The putative protein functions for DEGs were predicted based on the COG database. Among the 4381 DEGs, 2476 (56.52%) DEGs were annotated and classified into 24 specific COG categories according to the sequence homology (Additional file 3: Table S2). Within these COG categories, the “general function” (798, 32.23%), “carbohydrate transport and metabolism” (458, 18.50%), and “amino acid transport and metabolism” (440, 17.77%) were the most frequently represented functional clusters, followed by “inorganic ion transport and metabolism” (297, 12.00%), “secondary metabolites synthesis, transport and metabolism” (238, 9.61%), “transcription” (219, 8.84%), and “energy production and conversion” (218, 8.80%) (Fig. 3), indicating that complicated stress response mechanisms were induced in *A. oryzae* 112822 with nicotine treatment.

**Fig. 4:**
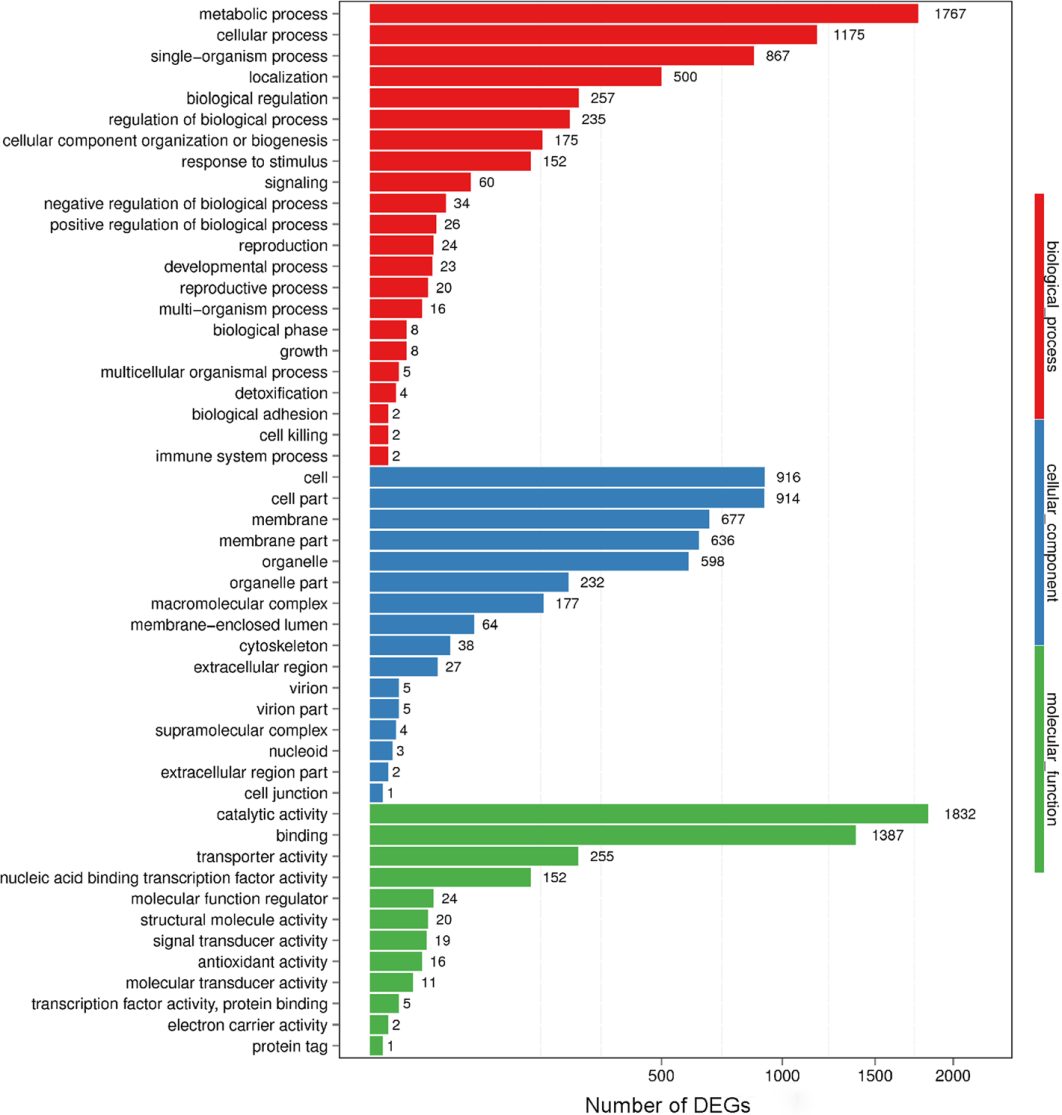
GO classification of DEGs. A total of 2908 DEGs were assigned to at least one GO term based on three categories: biological process (22 subcategories), cellular component (16 subcategories), and molecular function (12 subcategories). The x-axis indicates the number of DEGs, and the y-axis indicates the specific GO term

Based on the GO database, a total of 2908 (66.38%) DEGs were assigned to at least one GO term in three categories: biological process (2273), cellular component (1444), and molecular function (2512) (Additional file 3: Table S3). These three categories were further classified into 22, 16, and 12 subcategories, respectively. Among these GO categories, “metabolic process” (1767, 77.74%) and “cellular process” (1175, 51.69%) in biological process, “cell” (916, 63.43%) and “cell part” (914, 63.30%) in cellular component, and “catalytic activity” (1832, 72.93%) and “binding” (1387, 55.21%) in molecular function were the dominant categories compared with the whole transcriptome background (Fig. 4).

**Fig. 5:**
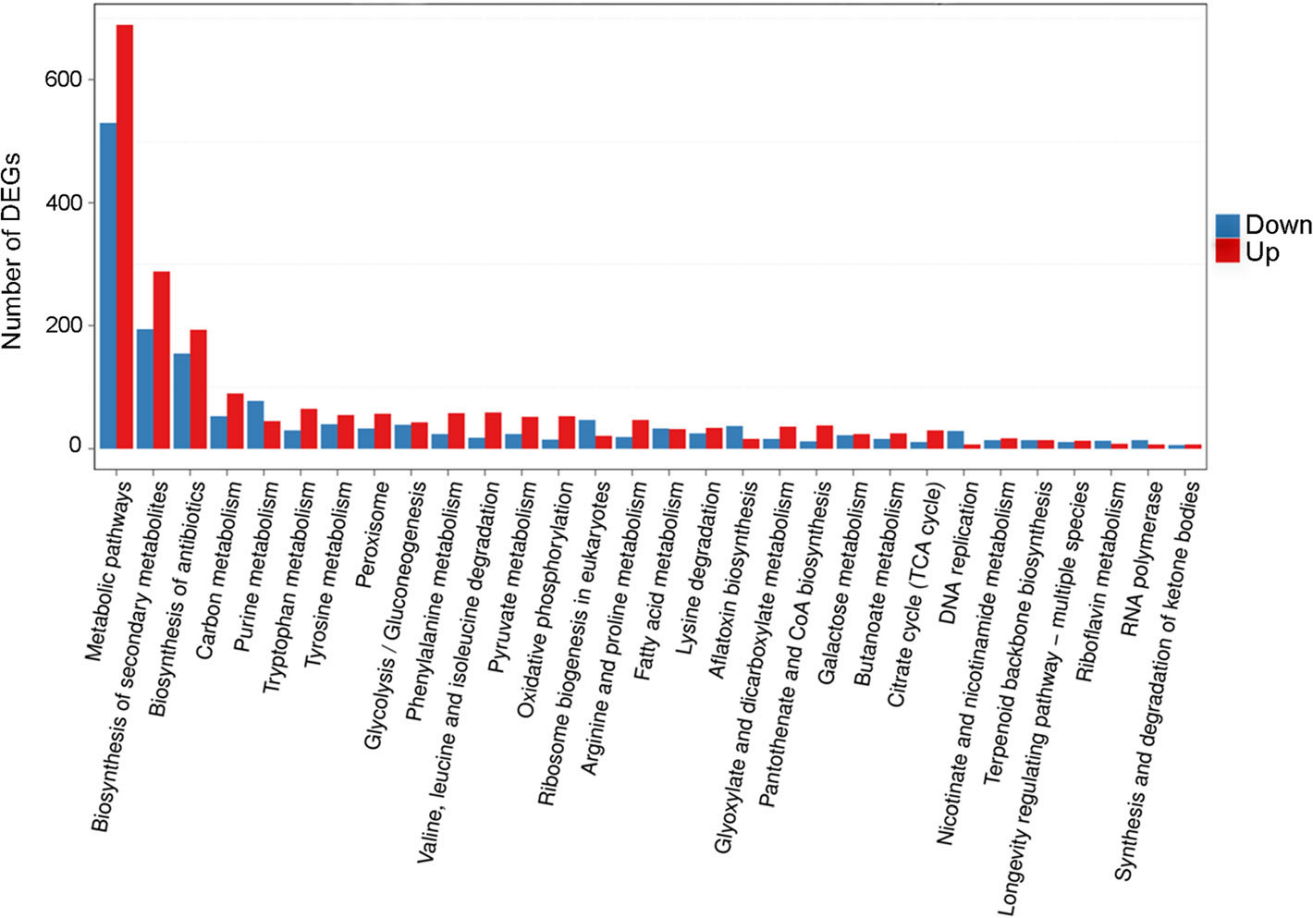
KEGG enrichment analyses of DEGs. The x-axis represents the most enriched pathways, the y-axis represents the number of DEGs. The red bars represent the up-regulated DEGs, and the blue bars indicate the down-regulated DEGs

To analyze the metabolic pathways associated with nicotine degradation, all the DEGs were searched against the KEGG database. As a result, 3269 (74.62%) DEGs were annotated into 124 KEGG pathways (Additional file 3: Table S4). The enriched KEGG pathways were dominantly represented by “metabolic pathways” (1219, 37.29%), “biosynthesis of secondary metabolites” (482, 14.74%), and “biosynthesis of antibiotics” (348, 10.65%). Besides, these pathways such as carbon metabolism, tryptophan metabolism, phenylalanine metabolism, valine, leucine and isoleucine degradation, pyruvate metabolism, oxidative phosphorylation, arginine and proline metabolism, and TCA cycle, were strongly induced by nicotine treatment (Fig. 5). These KEGG pathways were primarily associated with metabolic adjustment in response to nicotine exposure.

### Candidate genes involved in the demethylation pathway

The nicotine degradation activity of resting cells indicated the inducibility of nicotine degradation in *A. oryzae* 112822. Candidate genes involved in the nicotine demethylation pathway were proposed based on the DEGs analysis and the existing literature, including: i) 10 genes encoding cytochrome P450 monooxygenases (CYPs) which might be involved in *N*-demethylation of nicotine; ii) 1 gene encoding FAD-containing amine oxidase that potentially participates in dehydrogenation at the pyrrolidine-ring of nornicotine; iii) 2 genes encoding proteins involved in assisting the activation of molybdenum cofactor (Moco)-containing hydroxylase which might catalyze α-hydroxylation of the *sp*^*2*^–hybridized carbon of *N*-heterocyclic compound like NMN; iv) 1 gene encoding NADH-dependent and FAD-containing hydroxylase which might catalyze β-hydroxylation at the pyridine-ring of 2-HNMN and produce 2, 3-DHP (Scheme 1, Additional file 3: Table S5).

### Metabolic pathways involved in energy production

During nicotine detoxification, several metabolic pathways contributed to energy production were significantly induced, such as TCA cycle, oxidative phosphorylation, several types of amino acid metabolism, and fatty acid β-oxidation (FAO). Among the DEGs annotated in these metabolic pathways, 73.17% DEGs annotated in TCA cycle were up-regulated by nicotine treatment (Fig. 5). As the rate-limiting enzymes of TCA cycle, citrate synthase was 5.12-fold up-regulated, isocitrate dehydrogenase (IDH) was 2.16-fold up-regulated, and α-ketoglutarate dehydrogenase complex (α-KGDC) was 2.37-fold (E1 component) and 1.30-fold (E2 component) up-regulated (Additional file 3: Table S6). Averagely, 77.94% DEGs involved in oxidative phosphorylation were up-regulated in the Nic library (Fig. 5), and these DEGs functioned in each complex of electron transport chain were summarized individually (Additional file 3: Table S6). The reactions catalyzed by IDH, α-KGDC, and malate dehydrogenase in TCA cycle produce reducing equivalents (NADH) to drive ATP generation in oxidative phosphorylation pathway [38]. The amino acid metabolism pathways, including tryptophan metabolism, tyrosine metabolism, phenylalanine metabolism, valine, leucine and isoleucine degradation, arginine and proline metabolism, and lysine degradation, were significantly induced (Fig. 5). The genes encoding amidases, monoamine oxidases, and primary-amine oxidases that were typically involved in amino acid metabolism, were remarkably up-regulated (Additional file 3: Table S6). The catabolism of amino acid can also contribute to energy production. The precursor pyruvate and other intermediates, such as acetyl-coA, succinyl-coA, and succinate of TCA cycle, can be replenished by amino acid metabolism. Moreover, FAO was induced by the up-regulation of genes encoding acyl-CoA oxidase (from 1.45- to 5.06-fold), acyl-CoA dehydrogenase (2.23-fold), enoyl-CoA hydratase (from 2.51- to 3.45-fold), and acetyl-CoA acyltransferase (1.8-fold) (Additional file 3: Table S6). FAO is a crucial metabolic pathway for energy homoeostasis when glucose supply becomes limited [39], which also provides acetyl-coA for TCA cycle. These results suggested that increased energy was invested, most likely to meet the demand for nicotine detoxification and other defense mechanisms, which was consistent with the analysis of protein abundance levels of *P. putida* S16 and energy expenditure of *Spodoptera eridania* when exposed to nicotine [14, 40].

### Genes involved in oxidative stress responses

It has been reported that nicotine could generate a substantial level of reactive oxygen species (ROS) that form oxidative stress in rat cells [41]. ROS can cause damages to cell components, such as protein damage, DNA double-strand breakage, and lipid peroxidation. Antioxidant enzymes playing crucial role in ROS scavenging are generally induced in response to oxidative stress [42]. In the present study, antioxidant enzymes, such as superoxide dismutase (SOD) (1.15- to 1.33-fold), catalase (CAT) (2.34- to 3.25-fold), and peroxiredoxin (Prx) (2.17-fold) were found to be induced (Additional file 3: Table S7). In addition to activation of ROS scavenging, the repair mechanisms of oxidative damages were also triggered. The genes encoding DNA repair proteins, including Ku70 (3.71-fold), RAD50 (1.02- to 6.11-fold), Pol4 (1.80- to 4.68-fold), and Dnl4 (1.11- to 2.20-fold) were significantly up-regulated (Additional file 3: Table S7). The genes encoding heat shock proteins (Hsp), such as Hsp70 (3.19-fold) and Hsp90 (2.90-fold), were also up-regulated to assist in protein refolding and protecting cell against oxidative protein damage (Additional file 3: Table S7). Correspondingly, heat shock factors (HSF) were significantly up-regulated (4.07- to 9.56-fold) (Additional file 3: Table S7), which could act as an inducible transcriptional activator of genes encoding molecular chaperones [43]. Besides, the genes encoding thioredoxin 1 and thioredoxin reductase (TrxR) were 2.71- and 3.86-fold up-regulated respectively (Additional file 3: Table S7), suggesting that the thioredoxin system was induced to restore the intracellular redox homeostasis.

### Genes involved in phase II detoxification

Glutathione S-transferases (GSTs) belong to an important enzyme family involved in phase II detoxification of various xenobiotics. They can catalyze the conjugation of reduced glutathione (GSH) to the electrophilic center of xenobiotics for detoxification [44]. In the present study, four genes encoding GSTs were up-regulated from 1.67- to 3.51-fold in response to nicotine exposure (Additional file 3: Table S8), suggesting that they might participate in phase II detoxification of nicotine. Correspondingly, the genes encoding 5-oxoprolinase (12.0-fold), kynurenine 2-oxoglutarate transaminase (2.48- to 9.05-fold), and GSH synthase (2.19-fold) that involved in anabolism of GSH were also up-regulated (Additional file 3: Table S8). In addition to being an essential substrate for GSTs, GSH is also an effective antioxidant with thiol group as the reducing agent, and could prevent damages to important cellular components caused by ROS [45].

### Genes encoding transporters

For fungi, the overexpression of toxin efflux pumps, especially the ATP-Binding Cassette (ABC) transporters and the major facilitator superfamily (MFS) transporters, is a way to overcome the intracellular toxin accumulation [46]. In the present study, the ABC transporters PDR5 (1.05- to 10.06-fold) and SNQ2 (4.06-fold) were significantly up-regulated in *A. oryzae* 112822 in response to nicotine exposure. Besides, 12 genes encoding MFS toxin efflux pumps were also substantially up-regulated from 1.89- to 7.50-fold by nicotine treatment (Additional file 3: Table S9). These two types of toxin efflux pumps are classified into pleiotropic drug resistance (PDR) family and DHA1 family respectively, they are the major secondary transport systems that render resistance to drug and xenobiotics in organisms [46]. These findings indicated that ABC transporters and MFS transporters might be employed in extruding of cytotoxic metabolites generated during nicotine detoxification.

### The suppressed metabolic pathways

These metabolic pathways, such as purine metabolism, ribosome biogenesis, DNA replication, riboflavin metabolism, and RNA polymerase biosynthesis were strongly suppressed in *A. oryzae* 112822 in response to nicotine exposure (Fig. 5). These pathways are closely related to cellular activities like growth and reproduction. Nicotine detoxification was reported to impose a significant metabolic load and result in energy-limited growth for *Spodoptera eridania* [40], which may also apply to *A. oryzae* 112822 exposed to nicotine.

### Experimental validation

**Fig. 6:**
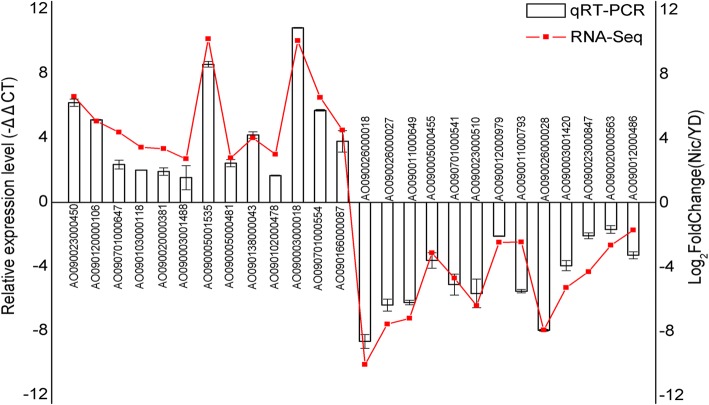
Expression profile validation of 26 selected DEGs by qRT-PCR analysis. Left y-axis represents the relative expression level determined by qRT-PCR, error bars indicate the average deviations of the three replicates. Right y-axis represents the transcript abundance based on DEGs analysis

**Fig. 7:**
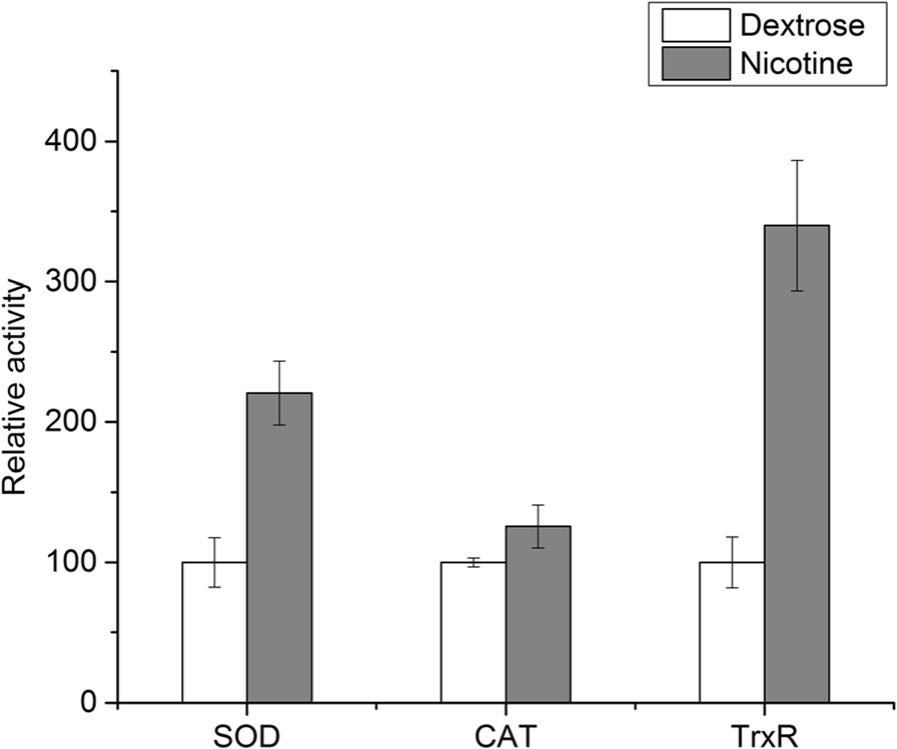
Enzymatic activity changes of oxidative stress markers related to nicotine degradation. Enzymatic activities of SOD, CAT, and TrxR were measured using their respective activity detection kit and performed via the microplate reader. The columns show activity levels of SOD, CAT, and TrxR in different media: white, dextrose-containing media; grey, nicotine-containing media. Bars indicate the standard deviation (*n* = 3)

In total, 13 up-regulated and 13 down-regulated genes were selected for quantitative real-time PCR (qRT-PCR) analysis to verify the differential expression between the YD and the Nic libraries. The genes encoding CYPs (AO090023000450, AO090120000106, AO090701000647), amine oxidases (AO090103000118, AO090020000381), molybdopterin molybdotransferase (AO090003001488), NADH-dependent flavoproteins (AO090005001535, AO090005000481), GSTs (AO090138000043, AO090102000478), Hsps (AO090003000018, AO090701000554), and DNA repair protein RAD50 (AO090166000087) were proved to be up-regulated by nicotine treatment through qRT-PCR, whereas the genes encoding aflatoxin B related enzymes (AO090026000018, AO090026000027), purine permeases (AO090011000649, AO090005000455), ribosome assembly protein (AO090701000541), ATP-dependent RNA helicase (AO090023000510), DNA replication licensing factors MCM4 (AO090012000979, AO090011000793), choline dehydrogenases (AO090026000028, AO090003001420, AO090023000847), RNA-dependent RNA polymerase (AO090020000563), and DNA-directed RNA polymerase (AO090012000486) were approved to have much lower transcriptional level in response to nicotine exposure through qRT-PCR. The qRT-PCR results were consistent with the transcript abundance changes obtained by DEGs analysis (Fig. 6), suggesting the reliability of transcriptome data. Moreover, the relative activities of three oxidative stress markers from nicotine-treated samples were analyzed compared with the control. As shown in Fig. 7, enzymatic activities of SOD, CAT, and TrxR increased 2.21-, 1.26-, and 3.40-fold by nicotine treatment, respectively. This result experimentally verified the oxidative stress responses related to nicotine tolerance and degradation in *A. oryzae* 112822.

## Discussion

The resting cells of *A. oryzae* 112822 did not display nicotine degradation activity unless the strain was treated with nicotine in advance, indicating the nicotine regulation on related gene expression. Hence, it was feasible to screen the genes and metabolic pathways associated with nicotine degradation in *A. oryzae* 112822 based on comparative transcriptome analysis. In the present study, we performed high-resolution RNA-seq to acquire a global view of the transcriptional regulation of *A. oryzae* 112822 exposed to nicotine. As a result, 16,818 transcripts those derived from 10,883 genes were detected from both the YD and the Nic libraries (Table 2). Among these genes, 4381 DEGs, namely 2291 up-regulated and 2090 down-regulated genes, were identified in the nicotine-treated samples (Fig. 2, Additional file 3: Table S1). Moreover, a large amount of biomarkers related to detoxification, oxidative stress response, DNA repair, heat shock response, and transporter were up-regulated in *A. oryzae* 112822 with nicotine treatment. These metabolic pathways related to energy production, such as TCA cycle, oxidative phosphorylation, and amino acid metabolisms were also strongly induced. All these results described complicated regulation networks involved in nicotine tolerance and degradation in *A. oryzae* 112822.

Nicotine exposure resulted in accumulating nicotine and its metabolites in *A. oryzae* 112822, oxidation of the pyrrolidine-ring and hydroxylation of the pyridine-ring of nicotine metabolites facilitate ring-cleaving and alleviate the toxicity of these compounds to a great extent [35]. In the present study, four groups of candidate genes that potentially participate in the demethylation pathway of nicotine degradation were proposed. Nicotine degradation in *A. oryzae* 112822 is initiated by nicotine *N*-demethylation that leads to nornicotine formation [35], which is consistent with the initial reaction catalyzed by CYP82E4 and CYP82E5v2 in *Nicotiana* sp. [30, 31] as well as the nicotine demethylation catalyzed by CYP2A6 and CYP2B6 in human hepatocyte [33]. CYPs constitute a large superfamily of heme-containing monooxygenases which distribute in a variety of organisms, they are generally involved in phase I detoxification of drugs and xenobiotics [44]. Accordingly, it was reasonably speculated that nicotine demethylation in *A. oryzae* 112822 might also be catalyzed by a specific CYP. A total of 142 CYPs are annotated in the genome of *A. oryzae* RIB40 [47]. Based on transcriptome analysis, 10 genes encoding CYPs were up-regulated in *A. oryzae* 112822 exposed to nicotine (Additional file 3: Table S5).

In the demethylation pathway of *A. oryzae* 112822, nornicotine is oxidized into myosmine by dehydrogenation at the pyrrolidine-ring, which is similar to the following reactions. In the pyridine/pyrrolidine pathway of bacteria and the demethylation pathway of tobacco and human, a step characterized by dehydrogenation of pyrrolidine-ring that results in formation of myosmine-like structure is essential for nicotine degradation [13, 14, 17, 21, 32, 34, 48] (Scheme 1). 6-Hydroxy-L-nicotine oxidase (6HLNO) catalyzes the dehydrogenation of 6-HN to produce 6-hydroxy-*N*-methylmyosmine which spontaneously hydrolyzes into 6-HPON in the pyridine pathway of *A. nicotinovorans* [13, 21]. 6-Hydroxynicotine oxidase (VppB) in the VPP pathway is an isoenzyme of 6HLNO, but they share low amino acid sequence identity of 24% [17]. In the pyrrolidine pathway, nicotine oxidoreductase (NicA2) of *P. putida* S16 and nicotine oxidase (NOX) of *P. putida* HZN6 catalyze the dehydrogenation of nicotine to produce NMM which spontaneously hydrolyzes into pseudooxynicotine [14, 48]. These pyrrolidine-ring dehydrogenases (6HLNO, VppB, NicA2, and NOX) all have a conserved FAD-binding motif (Rossman motif) at the *N*-terminal [17, 48], they belong to the amine oxidase family [13, 17, 21, 48] and were proved to be significantly regulated by nicotine [13, 14, 17, 48]. Here, an 8.78-fold up-regulated gene (AO090023000011) encoding FAD-containing amine oxidase (designated as DeII) was annotated in the transcriptome analysis (Additional file 3: Table S5). Multiple sequence alignment showed that DeII also had a conserved FAD-binding motif at the *N*-terminal. Phylogenetic analysis revealed that DeII had higher amino acid sequence identity with 6HLNO from the pyridine pathway (Additional file 4). Hence, we speculate that DeII might be responsible for the dehydrogenation of nornicotine to produce myosmine in the demethylation pathway of *A. oryzae* 112822.

The Moco-containing hydroxylases catalyze the hydroxylation of *sp*^*2*^-carbon at α-position of pyridine-ring, and play critical roles in the biodegradation of *N*-heterocycle compounds [14, 21, 27, 49–56]. In the demethylation pathway of *A. oryzae* 112822, the *sp*^*2*^-carbon at α-position of pyridine-ring of NMN also experiences hydroxylation [35] (Scheme 1). Although no gene encoding Moco-containing hydroxylase was up-regulated in the Nic library, in view of the up-regulation of two auxiliary proteins, molybdopterin molybdotransferase and Moco sulfurase (Additional file 3: Table S5), which are essential for Moco biosynthesis [57], we still speculate that a specific Moco-containing hydroxylase might be responsible for the α-hydroxylation of NMN in the demethylation pathway of *A. oryzae* 112822. Thus far, quite a few Moco-containing hydroxylases have been identified to catalyze the α-hydroxylation of pyridine-ring in the nicotine and nicotinic acid degradation pathways of bacteria, such as nicotine dehydrogenase (NDH_SML_) [49] and ketone dehydrogenase (KDH_SML_) [21] in the pyridine pathway of *A. nicotinovorans*, 3-succinoyl-pyridine monooxygenase (SpmABC) in the pyrrolidine pathway of *P. putida* S16 [14], nicotine hydroxylase (VppA_SL_) in the VPP pathway of *Ochrobactrum* sp*.* Strain SJY1 [27], nicotinic acid hydroxylases NicAB of *P. putida* KT2440 [50], NaDH_SLM_ of *Comamonas testosteroni* JA1 [51]_,_ NahAB_1_B_2_ of *Pusillimonas* sp. strain T2 [52], and Ndh_FSLM_ of *Eubacterium barkeri* [53] in the nicotinic acid degradation pathways (Additional file 5). NaDH_SLM_ can also hydroxylate 3-cyanopyridine to produce 3-cyano-6-hydroxypyridine [51]. Besides, the picolinic acid dehydrogenase (PaDH) of *Arthrobacter picolinophilus* DSM 20665^T^ [54], quinolinic acid dehydrogenase (QaDH) of *Alcaligenes* sp. strain UK21 [55], and isonicotinic acid dehydrogenase (INaDH) of *Mycobacterium* sp. INAl [56], can also catalyze the molybdenum-dependent pyridine-ring α-hydroxylation (Additional file 5).

In the demethylation pathway, 2, 3-DHP is identified as a pyridine-ring β-hydroxylation product after the oxidative decarboxylation of 2-HNMN [35] (Scheme 1), and the similar pyridine-ring β-hydroxylation has also been found in the nicotine and nicotinic acid degradation pathways of bacteria [17, 23, 26, 50, 58, 59]. As shown in Additional file 6, 2,6-dihydroxypyridine 3-hydroxylase (DHPH) catalyzes pyridine-ring β-hydroxylation of 2, 6-dihydroxypyridine (2, 6-DHP) and leads to 2, 3, 6-trihydroxypyridine (2, 3, 6-THP) production in the pyridine pathway of *A. nicotinovorans* [23]. HSP 3-monooxygenase (HspB) catalyzes a consecutive oxidative decarboxylation and hydroxylation reaction at the pyridine-ring β-position of HSP to produce 2, 5-dihydroxypyridine (2, 5-DHP) in the pyrrolidine pathway of *P. putida* S16 [26, 58]. VppD in the VPP pathway is an isoenzyme of HspB, and they share 62% amino acid sequence identity [17]. In addition, 6-hydroxynicotinic acid 3-monooxygenases (Pp_NicC and Bb_NicC) catalyze the similar reactions in the nicotinic acid degradation pathways of *P. putida* KT2440 and *Bordetella bronchiseptica* RB50, and also result in 2, 5-DHP formation using 6-hydroxynicotinic acid as substrate [50, 59]. The reaction catalyzed by DHPH is involved in hydroxylation of carbon atom that does not contain a substituent [23]. By contrast, HspB, VppD, Pp_NicC, and Bb_NicC catalyze a decarboxylative hydroxylation reaction, a relatively challenging process in which the carboxylate is removed and the hydroxyl is added at the same carbon atom [17, 26, 50, 59]. According to amino acid sequence alignment, these *N*-heterocyclic aromatic hydroxylases (HspB, VppD, Pp_NicC, and Bb_NicC) were characterized by three amino acid fingerprint motifs, namely GXGXXG, (D/E)XXIGADG, and GDA(A/C)H (Additional file 6), this type of hydroxylases contain FAD cofactor and rely on NADH as co-substrate [17, 23, 26, 50, 59]. DHPH displays amino acid sequence segments similar to these fingerprints with some substitutions, such as G → S in the first motif, D → N in the second motif, and H → V in the third motif [23]. Likewise, a 4.29-fold up-regulated gene (AO090102000087) encoding a putative protein (designated as DeIV) containing the three conserved fingerprint motifs was screened out (Additional file 3: Table S5), which might be responsible for the pyridine-ring β-hydroxylation of 2-HNMN in the demethylation pathway of *A. oryzae* 112822.

**Fig. 8:**
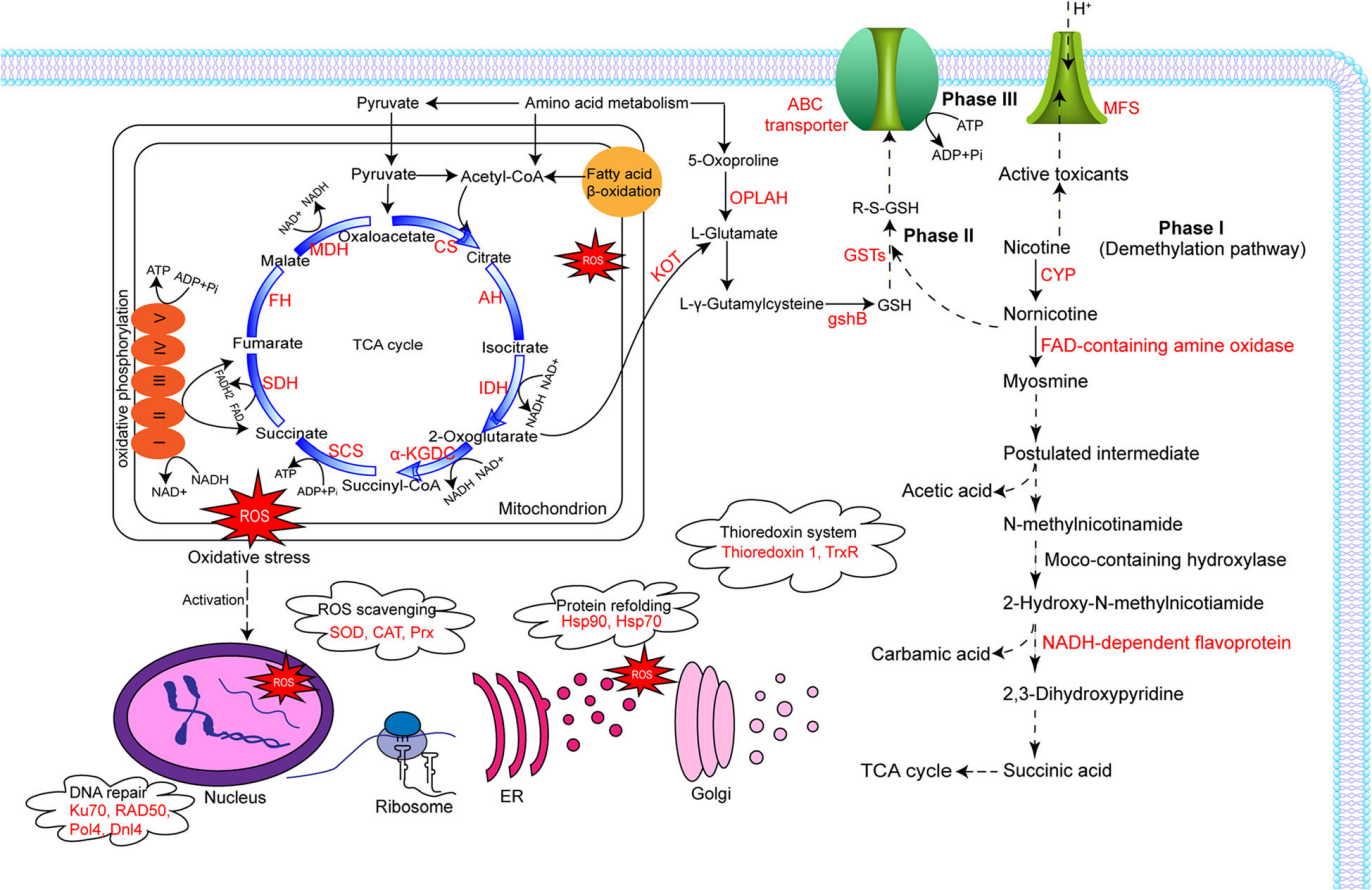
Proposed models of *A. oryzae* 112822 in response to nicotine exposure. The models were based on the results in the present study and in the existing literature (see Discussion for details). The proteins in red represent the induced enzymes. The dashed arrows represent the speculated information. CS, citrate synthase; AH, aconitate hydratase; IDH, isocitrate dehydrogenase; α-KGDC, α-ketoglutarate dehydrogenase complex; SCS, succinyl-CoA synthetase; SDH, succinate dehydrogenase; FH, fumarate hydratase; MDH, malate dehydrogenase; KOT, kynurenine 2-oxoglutarate transaminase; OPLAH, 5-oxoprolinase (ATP-hydrolysing); gshB, GSH synthase; GSTs, glutathione S-transferases; ABC, ATP-Binding Cassette; MFS, major facilitator superfamily; CYP, cytochrome P450 monooxygenase; Hsp, heat shock protein; SOD, superoxide dismutase; CAT, catalase; Prx, peroxiredoxin; Ku70, ATP-dependent DNA helicase 2 subunit 1; RAD50, DNA repair protein RAD50; Pol4, DNA polymerase IV; Dnl4, DNA ligase 4; ER, endoplasmic reticulum

The comparative transcriptome analysis highlighted a wide range of cellular processes of *A. oryzae* 112822 in response to nicotine treatment, which indicated that metabolic adjustment was pivotal for nicotine degradation. Hereby, nicotine tolerance and degradation mechanisms of *A. oryzae* 112822 were proposed in Fig. 8. Nicotine might be converted into less toxic and nontoxic metabolites through phase I detoxification (demethylation pathway), followed by phase II detoxification catalyzed by GSTs. These GSH-conjugated metabolites might be easily targeted to ABC transporters and exported via GSH group which could act as an affinity tag (phase III transport) [60]. Besides, the toxicants could also be exported by MFS transporters driven by electrochemical gradient [61]. These significantly induced pathways, such as TCA cycle, oxidative phosphorylation, and amino acid metabolism could provide energy to support nicotine detoxification, metabolites modification and export. The end-product of nicotine demethylation pathway, succinic acid, might also enter into TCA cycle for energy production. Increased flux of the amino acid metabolism and TCA cycle could provide L-glutamate as precursor for the enhanced anabolism of GSH, which could further function as the substrate of GSTs. In turn, these detoxification processes and enhanced energy metabolisms led to overproduction of ROS which formed oxidative stress within cells. Correspondingly, enzymatic antioxidants, including SOD, CAT, and Prx involved in ROS scavenging, were induced to regulate the intracellular ROS balance. Besides, thioredoxin system was also induced to restore the intracellular redox homeostasis. Other stress response proteins involved in DNA repair (Ku70, RAD50, Pol4, and Dnl4) and protein refolding (Hsp70 and Hsp90) were also triggered to promote nicotine tolerance.

## Conclusion

In the present study, we presented a global view of the transcriptional responses associated with nicotine degradation in *A. oryzae* 112822 through the comparative transcriptome analysis. Our results revealed that complicated regulation networks, involving detoxification, transport, and oxidative stress response accompanied by increased energy investment, were developed for nicotine tolerance and degradation in *A. oryzae* 112822. Moreover, candidate genes that potentially participate in phase I nicotine demethylation pathway were proposed, and they could be major targets for nicotine degradation study. This work provided the first insight into the metabolic regulation of nicotine degradation in filamentous fungi, and laid the foundation for further study of the nicotine demethylation pathway.

## Methods

### Fungal materials and samples collection

*A. oryzae* 112822 (CGMCC 3687) was isolated from tobacco leaves (General Cigarette Co. Ltd., China) [35]. Conidia were initially inoculated into an activated medium (10 g·L^− 1^ yeast extract, 20 g·L^− 1^ dextrose, 1 mM Mn^2+^, and pH 6.5) at 28 °C for 20 h. Then the mycelia were collected by filtration, equivalently transferred into dextrose-containing (2 g·L^− 1^ yeast extract, 10 g·L^− 1^ dextrose, 1 mM Mn^2+^, and pH 6.5) and nicotine-containing (2 g·L^− 1^ yeast extract, 2 g·L^− 1^ nicotine, 1 mM Mn^2+^, and pH 6.5) media, and cultured at 28 °C for another 16 h. For each culture, mycelia were collected for RNA preparation.

### Nicotine degradation by resting cells

Resting cells of *A. oryzae* 112822 were prepared as previously described [35]. Nicotine degradation assay was performed by adding 5 g of resting cells to 20 mL nicotine solution (0.35 g·L^− 1^) and incubating at 28 °C with shaking at 150 rpm. Reaction mixtures were sampled at regular intervals and analyzed by HPLC via an Agilent Technologies 1200 series (Agilent Technologies, USA) using an Agilent TC-C18 column (150 × 4.6 mm, Agilent Technologies, USA) and an UV detector operated at a wavelength of 254 nm.

### RNA extraction, cDNA preparation and sequencing

When the mycelia had grown in nicotine-containing media for 16 h, total RNA was extracted from three biological replicates of control (YD) and nicotine-treated (Nic) samples using RNAprep pure Plant kit (DP432, Tiangen, Beijing) according to the manufacturer’s instructions. The residual DNA was digested on-column by RNase-free DNase I during RNA purification. The quality of RNA was confirmed by NanoDrop-2000 spectrophotometer and Agilent 2100 bioanalyzer. For each sample, mRNA was isolated from 20 μg total RNA using magnetic beads with oligo (dT), cDNA libraries were constructed using the NEBNext Ultra RNA library prep kit (NEB, USA). The quality of cDNA libraries was controlled by the Agilent 2100 bioanalyzer and ABI StepOnePlus real-time PCR system. The libraries were sequenced on the Illumina HiSeq 4000 platform performed by BGI-Tech (Wuhan, China).

### Sequence mapping

Raw reads were generated through base calling, which transforms pyroluminescence intensity signals into nucleotide sequences. Raw reads were stringently filtered to obtain high-quality clean reads using SOAPnuke software (http://soap.genomics.org.cn/). The filtration was performed by removing adaptor sequences, reads containing more than 5%“N” (i.e., ambiguous bases in reads), and low quality reads in which more than 20% of the bases showed a q-value ≤15. The genome information of *A. oryzae* RIB40 (http://www.bio.nite.go.jp/dogan/Top) was used as the reference for reads mapping. Clean reads were mapped to reference genome with the efficient alignment aligner HISAT [62].

### Prediction of novel transcripts

Transcripts were reconstructed using StringTie [63] and integrated by Cuffmerge [64] for all the cDNA libraries, the integrated information was aligned against reference annotation information for novel transcripts prediction. The protein coding potential of novel transcripts was predicted by CPC [65], and the intact reference sequence information was formed by adding the novel transcripts with protein coding potential to the reference sequence of *A. oryzae* RIB40. Subsequent analysis was based upon the reconstructed reference sequence.

### Functional annotation and DEG identification

Functional annotation of genes was conducted by searching against the Nr database using BLAST program with E-value < 10^− 5^. For further functional characterization, the gene sequences were aligned against the COG database to predict the putative protein functions. The Blast2GO program [66] was used to acquire the GO annotation according to biological process, cellular component, and molecular function ontologies. Metabolic pathway assignment was conducted by searching against the KEGG database. The gene expression levels were quantified by RSEM package [37], and the DEGs between the YD and Nic libraries were identified using DEseq2 method [67] according to the criterion of |log_2_ Fold Change| ≥1 and Padj ≤0.05. The annotation information for DEGs was extracted and classified.

### qRT-PCR validation

To experimentally validate the DEGs analysis, 26 DEGs were selected to be quantified by qRT-PCR. Reverse transcription reactions with approximately 4.5 μg RNA for each sample were performed using RevertAid first strand cDNA synthesis kit (Thermo Scientific, USA) following the manufacturer’s instructions. The qRT-PCR primers were listed in Additional file 3: Table S10. β-actin gene (AO090701000065) was used as the internal control. qRT-PCR was performed on a Roche Diagnostics Light Cycler 480 (Roche Applied Science) using THUNDERBIRD SYBR® qPCR Mix (Toyobo Co. Ltd., China). The amplification reactions were performed following the PCR program: first denaturation at 95 °C for 2 min, then 40 cycles of denaturation at 95 °C for 10 s, annealing at 60 °C for 30 s, and extension at 80 °C for 30 s. This experiment was performed with three biological replicates, and the data were analyzed using Rotor-Gene Q series software (Qiagen, Germany). The relative expression levels of the selected genes normalized to β-actin were calculated using 2^-ΔΔCt^ method [68].

### Activity detection of oxidative stress markers

Cell lysate was prepared by grinding of mycelia with liquid nitrogen and adding buffer from the respective activity detection kit (Solarbio., China). After centrifugation at 12,000 rpm at 4 °C for 10 min, the supernatant was used for detection of enzymatic activities of SOD, CAT, and TrxR according to the manufacturer’s instructions via the microplate reader in 96-well plates. The relative activities of nicotine-treated sample were analyzed compared with the control.

## Additional files

Additional file 1: The coverage of the clean reads for transcripts. a The coverage counted for YD libraries of three biological replicates. b The coverage counted for Nic libraries of three biological replicates. (TIF 433 kb)

Additional file 2: Venn diagrams analysis for YD (a) and Nic (b) libraries. (TIF 346 kb)

Additional file 3: **Table S1.** DEGs between the YD and Nic libraries. **Table S2.** COG classification of DEGs. **Table S3.** GO classification and enrichment analysis of DEGs. **Table S4.** KEGG pathway annotation of DEGs. **Table S5.** Candidate genes involved in the demethylation pathway of nicotine degradation in *A. oryzae* 112822. **Table S6.** DEGs associated with energy production in *A. oryzae* 112822 exposed to nicotine. **Table S7.** DEGs encoding enzymes involved in oxidative stress responses. **Table S8.** DEGs encoding enzymes involved in phase II detoxification. **Table S9.** DEGs encoding transporters involved in efflux of nicotine and its metabolites. **Table S10.** Information for the primers used in the qRT-PCR analysis. (XLSX 1155 kb)

Additional file 4: Phylogenetic analysis and multiple sequence alignment of DeII and related pyrrolidine-ring dehydrogenases. The phylogenetic tree was constructed using the neighbor-joining method. The GenBank accession numbers are shown in parentheses. The length of the lines is proportional to the genetic distance between these proteins. The bar represents 0.1 amino acid substitution per site. (TIF 397 kb)

Additional file 5: Pyridine-ring α-hydroxylation catalyzed by Moco-containing hydroxylases. a Pyridine-ring α-hydroxylation occurred in pyridine derivatives metabolism. PaDH, picolinic acid dehydrogenase; QaDH, quinolinic acid dehydrogenase; INaDH, isonicotinic acid dehydrogenase. **b** Molecular architecture of several Moco-containing hydroxylases. NDH_SML_ (GenBank accession numbers CAA53087, CAA53086, and CAA53088), nicotine dehydrogenase from *A. nicotinovorans*; KDH_SML_ (WP_016359457, WP_016359456, and WP_016359451), ketone dehydrogenase from *A. nicotinovorans*; SpmABC (AEJ14617 and AEJ14616), 3-succinoyl-pyridine monooxygenase from *P. putida* S16; Ndh_FSLM_ (ABC88396, ABC88397, ABC88398, and ABC88399), nicotinic acid dehydrogenase from *Eubacterium barkeri*; NicAB (NP_746077 and NP_746078), nicotinic acid dehydrogenase from *P. putida* KT2440; NaDH_SLM_ (ACA29530, ACA29531, and ACA29532), nicotinic acid dehydrogenase from *Comamonas testosteroni* JA1; NahAB_1_B_2_ (OXR49108, OXR49107, and OXR49110), nicotinic acid dehydrogenase from *Pusillimonas* sp. strain T2; and VppA_SL_ (AIH15807 and AIH15806), nicotine hydroxylase from *Ochrobactrum* sp. strain SJY1. The letters depicted below the proteins indicate the subunit names of the corresponding proteins. (TIF 549 kb)

Additional file 6: Pyridine-ring β-hydroxylation catalyzed by NADH-dependent and FAD-containing hydroxylases. a Pyridine-ring β-hydroxylation occurred in the nicotine and nicotinic acid degradation pathways of bacteria. **b** Multiple sequence alignment of DeIV and other FAD-containing *N*-heterocyclic aromatic hydroxylases. DHPH (GenBank accession number YP_007988763), 2, 6-dihydroxypyridine 3-hydroxylase from *A. nicotinovorans*; HspB (ADN26547), HSP 3-monooxygenase from *P. putida* S16; VppD (AIH15770), HSP 3-monooxygenase from *Ochrobactrum* sp*.* Strain SJY1; Pp_NicC (NP_746074), 6-hydroxynicotinic acid 3-monooxygenase from *P. putida* KT2440; Bb_NicC (WP_010926295), 6-hydroxynicotinic acid 3-monooxygenase fron *B. bronchiseptica* RB50. The multiple alignment was performed using COBLAST. The identical regions were shaded in black, and the similar regions were shaded in gray. Moreover, the highly conserved fingerprint motifs were marked by lines above, and the substituted amino acids in similar sequence segments of DHPH were marked by asterisks. (TIF 1866 kb)

## Abbreviations

2, 3-DHP: 2, 3-dihydroxypyridine
2, 5-DHP: 2, 5-dihydroxypyridine
2-HNMN: 2-hydroxy-*N*-methylnicotinamide
6HLNO: 6-hydroxy-L-nicotine oxidase
6-HN: 6-hydroxy-L-nicotine
6-HPON: 6-hydroxypseudooxynicotine
ABC: ATP-Binding Cassette
CAT: Catalase
COG: Clusters of Orthologous Groups
CPC: Coding Potential Calculator
CYPs: Cytochrome P450 monooxygenases
DEGs: Differentially expressed genes
DHA1: Drug: H^+^ Antiporter-1 family
DHPH: 2, 6-dihydroxypyridine 3-hydroxylase
Dnl4: DNA ligase 4
FAD: Flavin adenine dinucleotide
FAO: Fatty acid β-oxidation
GO: Gene Ontology
GSH: Glutathione
GSTs: Glutathione S-transferases
HISAT: Hierarchical indexing for spliced alignment of transcripts
HPLC: High performance liquid chromatography
HSF: Heat shock factor
Hsp: Heat shock protein
HSP: 6-hydroxy-3-succinoyl-pyridine
HspB and VppD: HSP 3-monooxygenase
IDH: Isocitrate dehydrogenase
KEGG: Kyoto Encyclopedia of Genes and Genomes
Ku70: ATP-dependent DNA helicase 2 subunit 1
MFS: Major facilitator superfamily
Moco: Molybdenum cofactor
NADH: Reduced form of nicotinamide-adenine dinucleotide
NCBI: National Center for Biotechnology Information
NDMs: Nicotine-degrading microorganisms
NicA2: Nicotine oxidoreductase
NicC: 6-hydroxynicotinic acid 3-monooxygenase
NMM: *N*-methylmyosmine
NMN: *N*-methylnicotinamide
NOX: nicotine oxidase
Nr: non-redundant
Padj: Adjusted *P*-value
PDR: pleiotropic drug resistance
Pol4: DNA polymerase IV
Prx: peroxiredoxin
qRT-PCR: quantitative real-time PCR
RAD50: DNA repair protein RAD50
ROS: Reactive oxygen species
RSEM: RNA-Seq by expectation maximization
SOD: Superoxide dismutase
TCA: Tricarboxylic acid
TrxR: Thioredoxin reductase
VPP: Variant of pyridine and pyrrolidine
VppB: 6-hydroxynicotine oxidase
α-KGDC: α-ketoglutarate dehydrogenase complex
