# Supplementary figures and images for: Transcriptome analysis of genes and metabolic pathways associated with nicotine degradation in Aspergillus oryzae 112822
Source: BMC Genomics. 2019 Jan 24;20:86. doi: 10.1186/s12864-019-5446-2 (PMC6346535; doi:10.1186/s12864-019-5446-2)

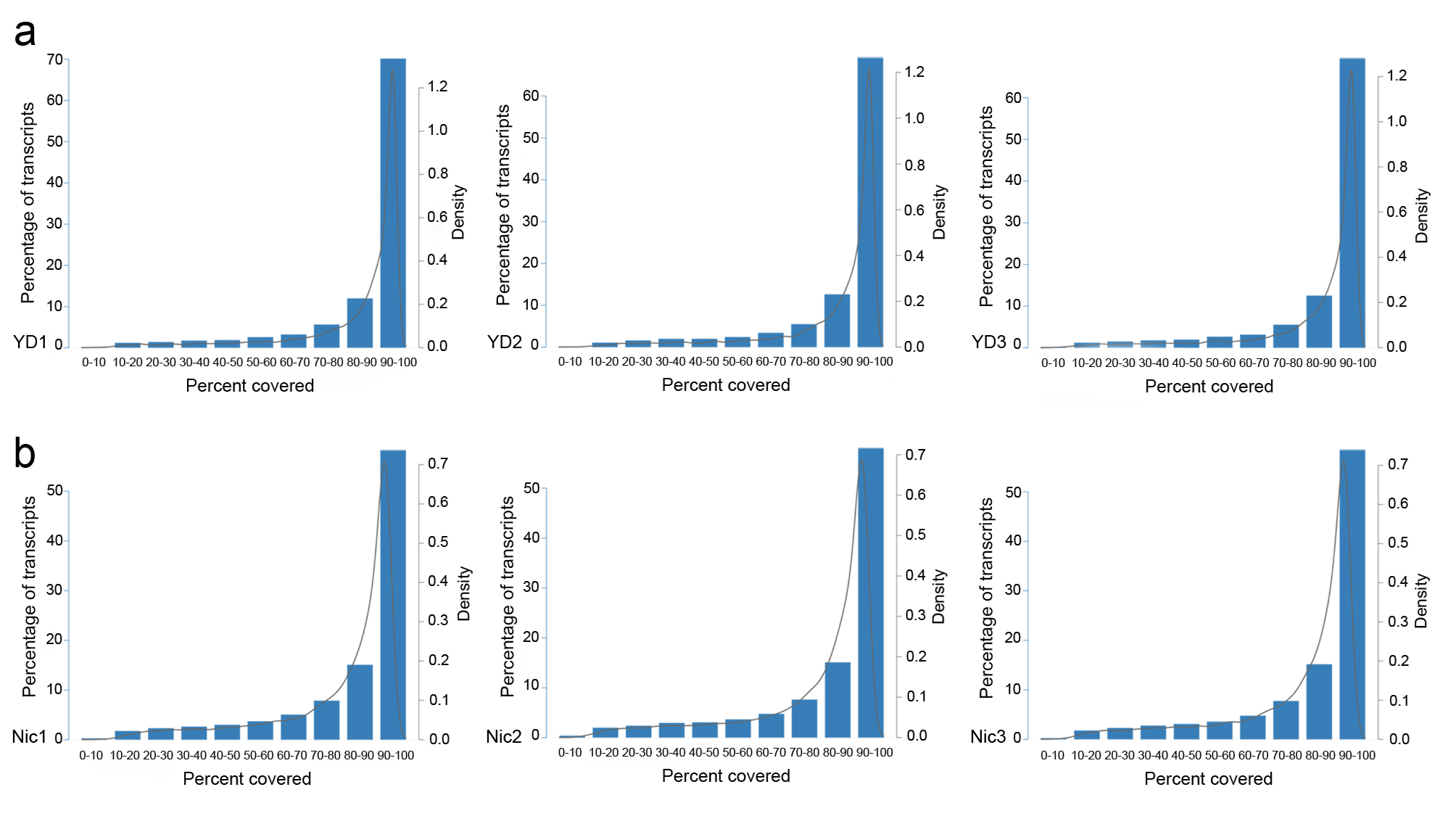

Supplement: Supplementary file 1 — The coverage of the clean reads for transcripts. a The coverage counted for YD libraries of three biological replicates. b The coverage counted for Nic libraries of three biological replicates. (TIF 433 kb) [file 12864_2019_5446_MOESM1_ESM.tif]

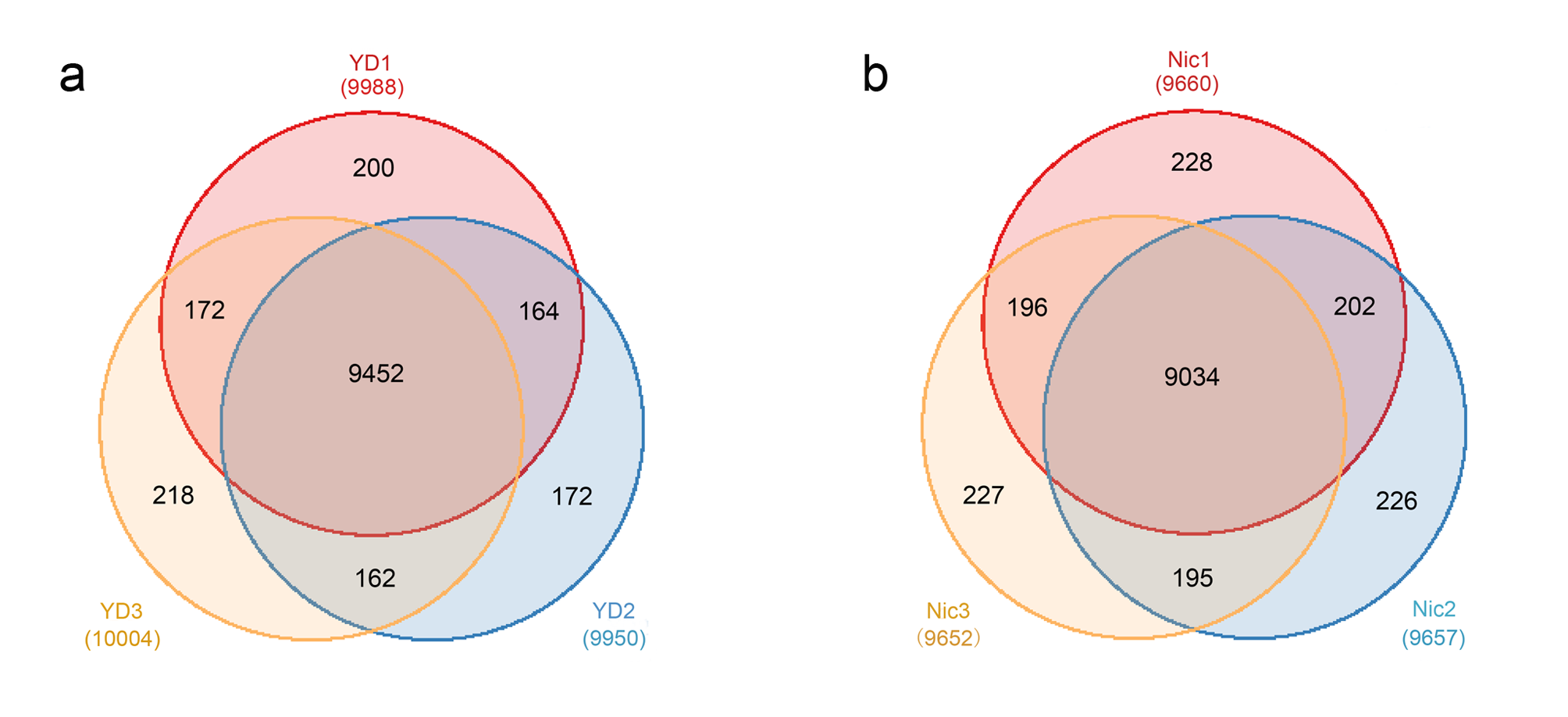

Supplement: Supplementary file 2 — Venn diagrams analysis for YD (a) and Nic (b) libraries. (TIF 346 kb) [file 12864_2019_5446_MOESM2_ESM.tif]

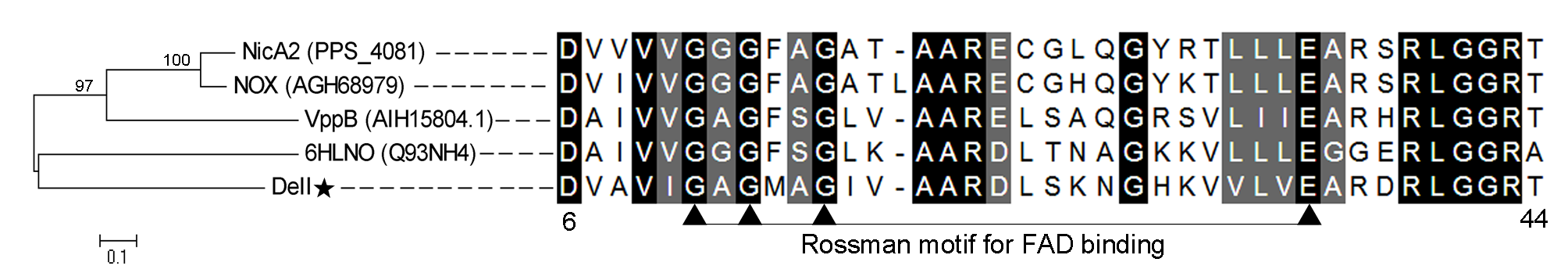

Supplement: Supplementary file 4 — Phylogenetic analysis and multiple sequence alignment of DeII and related pyrrolidine-ring dehydrogenases. The phylogenetic tree was constructed using the neighbor-joining method. The GenBank accession numbers are shown in parentheses. The length of the lines is proportional to the genetic distance between these proteins. The bar represents 0.1 amino acid substitution per site. (TIF 397 kb) [file 12864_2019_5446_MOESM4_ESM.tif]

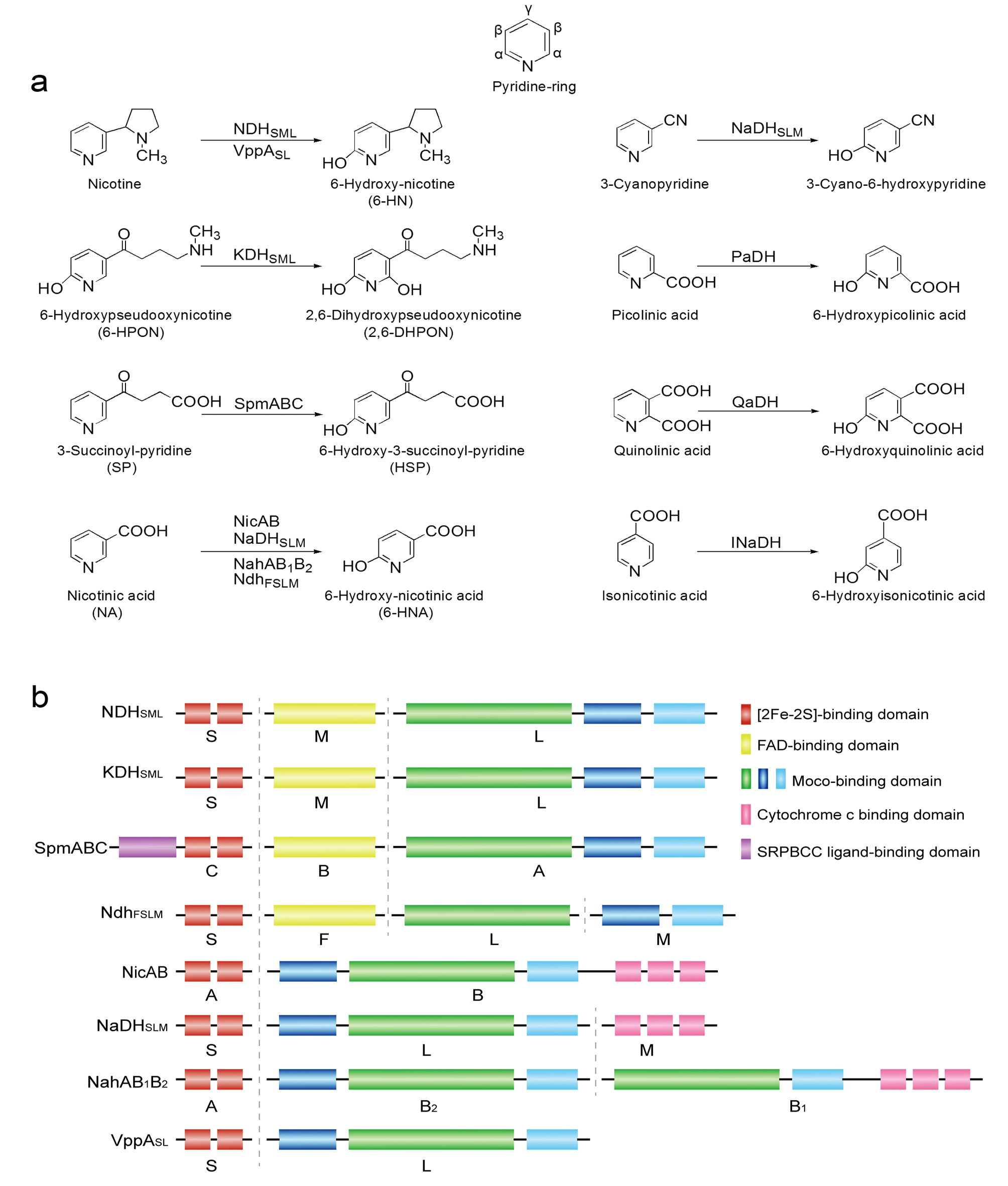

Supplement: Supplementary file 5 — Pyridine-ring α-hydroxylation catalyzed by Moco-containing hydroxylases. a Pyridine-ring α-hydroxylation occurred in pyridine derivatives metabolism. PaDH, picolinic acid dehydrogenase; QaDH, quinolinic acid dehydrogenase; INaDH, isonicotinic acid dehydrogenase. b Molecular architecture of several Moco-containing hydroxylases. NDHSML (GenBank accession numbers CAA53087, CAA53086, and CAA53088), nicotine dehydrogenase from A. nicotinovorans; KDHSML (WP_016359457, WP_016359456, and WP_016359451), ketone dehydrogenase from A. nicotinovorans; SpmABC (AEJ14617 and AEJ14616), 3-succinoyl-pyridine monooxygenase from P. putida S16; NdhFSLM (ABC88396, ABC88397, ABC88398, and ABC88399), nicotinic acid dehydrogenase from Eubacterium barkeri; NicAB (NP_746077 and NP_746078), nicotinic acid dehydrogenase from P. putida KT2440; NaDHSLM (ACA29530, ACA29531, and ACA29532), nicotinic acid dehydrogenase from Comamonas testosteroni JA1; NahAB1B2 (OXR49108, OXR49107, and OXR49110), nicotinic acid dehydrogenase from Pusillimonas sp. strain T2; and VppASL (AIH15807 and AIH15806), nicotine hydroxylase from Ochrobactrum sp. strain SJY1. The letters depicted below the proteins indicate the subunit names of the corresponding proteins. (TIF 549 kb) [file 12864_2019_5446_MOESM5_ESM.tif]

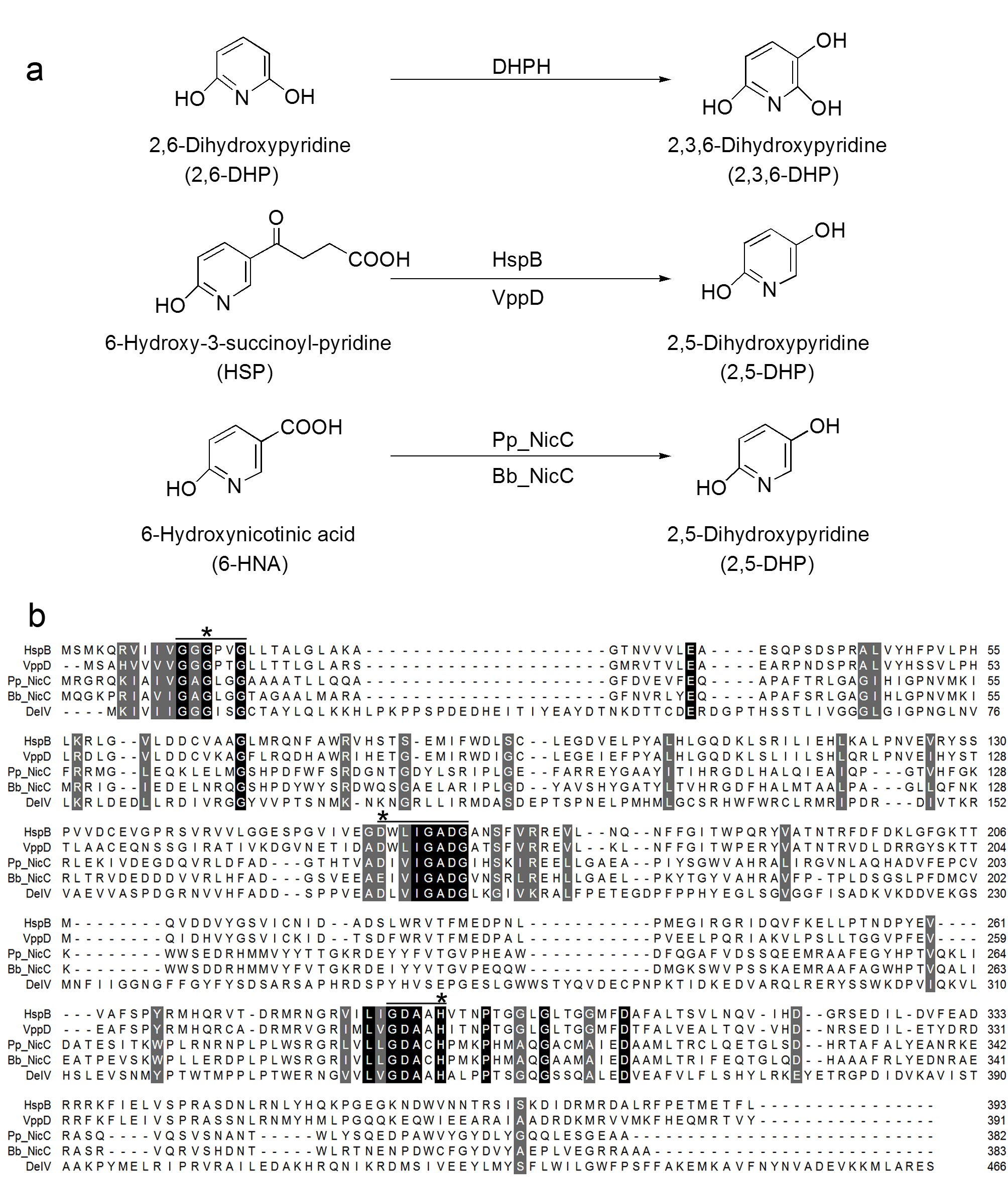

Supplement: Supplementary file 6 — Pyridine-ring β-hydroxylation catalyzed by NADH-dependent and FAD-containing hydroxylases. a Pyridine-ring β-hydroxylation occurred in the nicotine and nicotinic acid degradation pathways of bacteria. b Multiple sequence alignment of DeIV and other FAD-containing N-heterocyclic aromatic hydroxylases. DHPH (GenBank accession number YP_007988763), 2, 6-dihydroxypyridine 3-hydroxylase from A. nicotinovorans; HspB (ADN26547), HSP 3-monooxygenase from P. putida S16; VppD (AIH15770), HSP 3-monooxygenase from Ochrobactrum sp. Strain SJY1; Pp_NicC (NP_746074), 6-hydroxynicotinic acid 3-monooxygenase from P. putida KT2440; Bb_NicC (WP_010926295), 6-hydroxynicotinic acid 3-monooxygenase fron B. bronchiseptica RB50. The multiple alignment was performed using COBLAST. The identical regions were shaded in black, and the similar regions were shaded in gray. Moreover, the highly conserved fingerprint motifs were marked by lines above, and the substituted amino acids in similar sequence segments of DHPH were marked by asterisks. (TIF 1866 kb) [file 12864_2019_5446_MOESM6_ESM.tif]
